# Short-cut nitrogen removal from high-strength ammonia wastewater in a sequencing batch biofilm reactor: roles of NO and its production mechanism

**DOI:** 10.3389/fmicb.2025.1653308

**Published:** 2025-12-04

**Authors:** Junkai Zhao, Ju Zhang, Heng Yu, Wenjuan Yang, Jianqiang Zhao, Shuhan Lei, Jie Yang

**Affiliations:** 1 College of Chemistry and Chemical Engineering, Xi’an Shiyou University, Xi’an, Shaanxi, China; 2 Shaanxi Province Key Laboratory of Environmental Pollution Control and Reservoir Protection Technology of Oilfields, Xi’an, Shaanxi, China; 3 School of Water and Environment, Chang’an University, Xi’an, Shaanxi, China; 4 Key Laboratory of Subsurface Hydrology and Ecological Effect in Arid Region (Chang’an University), Ministry of Education, Xi’an, Shaanxi, China; 5 Shaanxi Anliansheng Environmental Protection Technology Co., Ltd., Xi’an, Shaanxi, China

**Keywords:** short-cut nitrogen removal, high-strength ammonia wastewater, nitric oxide, sequencing batch biofilm reactor, role and mechanism

## Abstract

Nitric oxide (NO) is a key intermediate in the biological nitrogen removal process. However, its role and production mechanism is still not fully understood. In this study, a sequencing batch biofilm reactor (SBBR) was used to study the short-cut nitrogen removal from high-strength ammonia wastewater and NO production mechanism. The ammonia concentration in SBBR was 1,000 mg-N/L, with a carbon-nitrogen ratio of 5, the simultaneous partial nitrification and denitrification efficiency reached 66.42%, while the average total inorganic nitrogen removal efficiency was 83.37 ± 6.93%. Microbial community analysis showed the vital role of functional bacteria such as *Thauera, Stappia,* and *Nitrosomonas* in the short-cut nitrogen removal process. The accumulation of NO occurred mainly under aerobic conditions, with the highest NO concentration of 0.19 mg-N/L. NO accumulation was mainly attributed to the incomplete oxidation of hydroxylamine, nitrifier denitrification and heterotrophic denitrification. Synergistic inhibition of nitrite-oxidizing bacteria by NO with free ammonia and free nitrous acid contributed to rapid establishment of partial nitrification and long-term stability of the process. The present study provides novel insights into the underlying mechanisms mediating the inhibition of nitrite-oxidizing bacteria.

## 1 Introduction

Biological treatment is an effective method for removing nitrogen pollutants from water. This method is widely used in urban sewage treatment plants. However, the conventional nitrification–denitrification process requires high-intensity aeration and additional supply of organic carbon, which lead to higher operational costs. The partial nitrification and denitrification (PND) process controls the nitrification reaction in the nitrite stage, followed by denitrification. Compared to the nitrification–denitrification process, 25% of aeration is saved in the nitrification stage, while organic carbon consumption reduced by nearly 40% in the denitrification stage of PND (Jia et al., 2022; Wang et al., 2010). The process also shows better nitrogen removal efficiency during the treatment of ammonia-rich wastewater.

Nitric oxide (NO) is both a toxic molecule and a key intermediate involved in the microbial nitrogen cycle (Bowman et al., 2011). During biological nitrogen removal, NO is produced through multiple pathways, including incomplete oxidation of hydroxylamine (NH₂OH), nitrifier denitrification, and heterotrophic denitrification (Zhao et al., 2022b). Free ammonia (FA)-rich and free nitrous acid (FNA)-rich environment inhibits the activity of NO reductase (*Nor*), leading to excessive NO accumulation (Wang et al., 2019a). Microorganisms generally have a low level of tolerance to NO toxicity. High concentrations of nitrite can lead to higher NO accumulation in the reactor, with NO concentration reaching the level of milligrams per liter (Wang et al., 2019b; Xie et al., 2021). Due to its intrinsic toxicity, the accumulated NO inhibits the activity of *Nor* and nitrous oxide reductase (*Nos*), leading to failure of the wastewater treatment system and emission of a large amount of greenhouse gasses, such as nitrous oxide (N_2_O) — a potent greenhouse gas (Wang et al., 2019a).

Interestingly, recent studies indicate that at appropriate concentrations, NO can selectively inhibit nitrite-oxidizing bacteria (NOB) without significantly harming ammonia-oxidizing bacteria (AOB), thereby facilitating the rapid establishment of partial nitrification and sustaining its long-term stability (Lei et al., 2023; Xie et al., 2021). While excessive NO can exert detrimental effects on biological nitrogen removal systems, an appropriate concentration of NO may have a beneficial role, suggesting that its function in PND systems is more complex than previously recognized. Therefore, it is essential to further elucidate the generation and accumulation characteristics of NO under ammonia-rich wastewater conditions and to determine whether its concentration can positively influence the enrichment of key functional microbial populations (e.g., *Nitrosomonas*, *Thauera*, and *Stappia*) as well as enhance process performance, which is crucial for optimizing PND operation.

In this study, PND process was adopted for treatment of high-strength ammonia wastewater. The pollutant removal performance of this process was assessed in a sequencing batch biofilm reactor (SBBR) by measuring pH and concentrations of ammonia, nitrite, nitrate, dissolved oxygen (DO), dissolved NO and N_2_O. Furthermore, production mechanism and role of NO were determined by analyzing the transformation characteristics of pollutants and the changes in the structure of microbial community. This study elucidates the production characteristics of NO during the treatment of high-strength ammonia wastewater. Importantly, the synergistic inhibitory effects of NO with FA and FNA not only facilitated the rapid establishment of PND but also sustained long-term process stability. These findings provide new mechanistic insights into the performance optimization of PND process for treating ammonia-rich wastewater.

## 2 Materials and methods

### 2.1 Sludge and sewage

The seed sludge used in this study came from the activated sludge with nitrification and denitrification ability, domesticated in the laboratory. The biofilm carrier used for SBBR was a polyester fiber composite filler (Specification: 1.5D, Sichuan Xingmeixin Environmental Protection Technology Group Co. Ltd.), which could form a film-like bio-sludge on the carrier after sludge seeding. The mixed liquor suspended solids was 3,800 mg/L. The settling velocity (SV_30_) was 23%. Because the biofilm grows attached to the carrier and is not easily washed out, its effective sludge retention time (SRT) is generally much longer than that of conventional suspended activated sludge systems. In this study, the effective SRT was approximately 50–55 days.

The synthetic wastewater was used as the influent, which mainly included NH_4_HCO_3_ (5,643 mg/L), C_6_H_12_O_6_ (4,688 mg/L) and NaHCO_3_ (12,000 ~ 14,000 mg/L) (the mass of NaHCO_3_ was adjusted as required), as well as MgSO_4_·7H_2_O 300 mg/L, KH_2_PO_4_ 60 mg/L, CaCl_2_ 150 mg/L and 1 mL of trace element stock solution per liter of feed. The trace element solution used in this study contained the following components: H₃BO₃ (50 mg/L), CuCl₂ (30 mg/L), ZnCl₂ (50 mg/L), (NH₄)₆Mo₇O₂·4H₂O (50 mg/L), MgSO₄·7H₂O (500 mg/L), CoCl₂·6H₂O (50 mg/L), AlCl₃ (50 mg/L), NiCl₂ (50 mg/L), and concentrated hydrochloric acid (1 mL/L). The theoretical ammonia concentration of influent was 1,000 mg-N/L, with a carbon-nitrogen ratio (C/N) of 5 (mg COD/mg N). Glucose was used as the carbon source.

### 2.2 Reactor operation and experimental procedure

A reactor with an effective volume of 2 L was used to study the nitrogen removal performance of activated sludge biofilm for treating high-strength ammonia wastewater. The reactor was kept in a uniform state by magnetic stirring. The reactor was operated for two cycles per day, with each cycle of 720 min. A cycle consisted of 6 min of feeding, 60 min of anoxic treatment, 600 min of aerobic aeration, 30 min of settling, 6 min of decanting, and 18 min of idle state. A prolonged aerobic aeration stage was designed in this study to ensure stable ammonium oxidation under high-ammonia loading, thereby effectively promoting efficient nitrogen removal in SBBR system.

**Figure 1:**
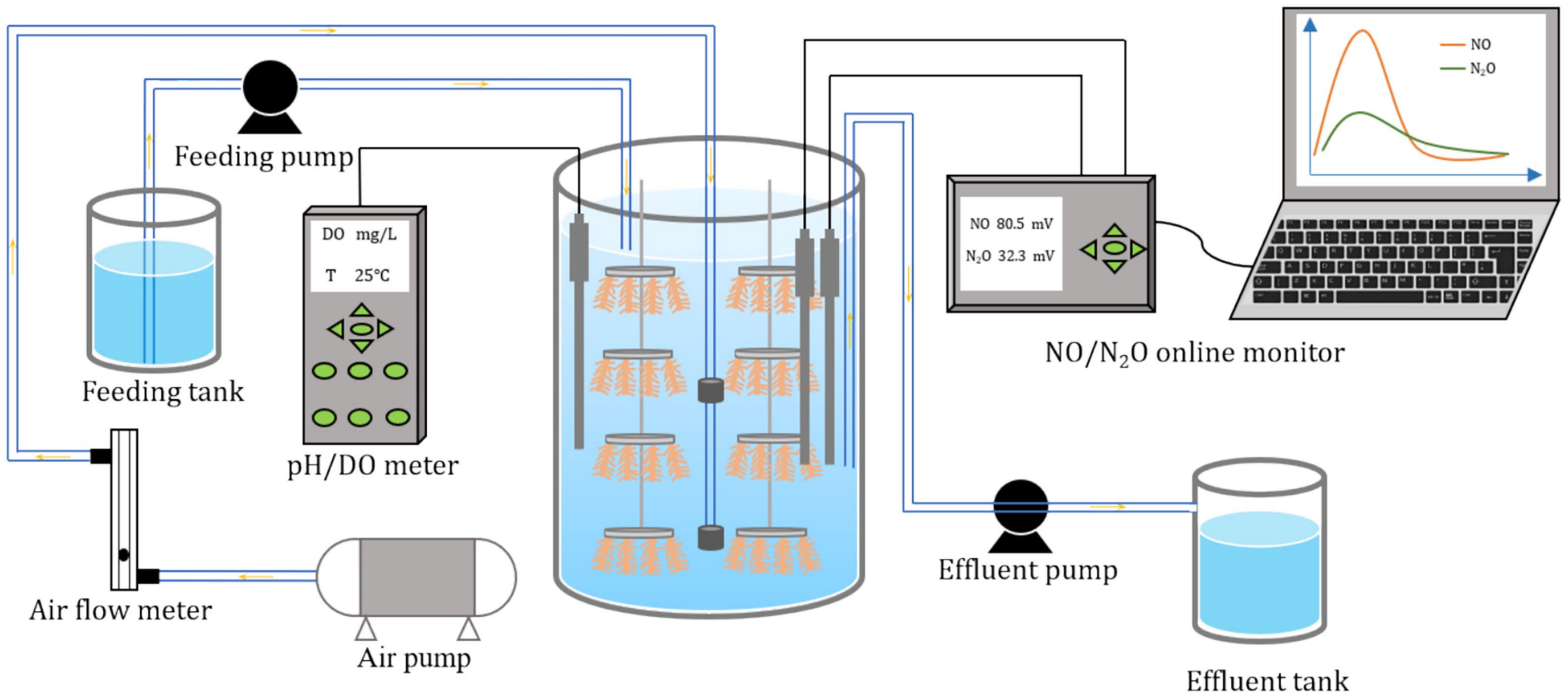
Schematic representation of the SBBR.

The reactor was operated at a controlled temperature of 25 ± 1 °C, with an initial pH of 7.5–8.8. During days 1–27, the discharge ratio was 20%, and the hydraulic retention time (HRT) was 60 h. From days 28–142, the discharge ratio was decreased to 10%, and HRT was extended to 120 h. The initial DO setpoint was approximately at 3 ± 0.5 mg/L. To maintain partial nitrification, given that the aerobic aeration time within each cycle was kept constant, the aeration rate needed to be adjusted according to DO concentration and the ammonia oxidation rate to ensure that DO concentration remained at the designated level (Figure 1).

The SBBR was operated in three phases. In Phase I (days 1 ~ 27), the reactor was started with a discharge ratio of 20%, with initial theoretical ammonia concentration (after feeding) of 200 mg-N/L and chemical oxygen demand (COD) of 1,000 mg/L. In Phase II (days 28 ~ 74), the discharge ratio was adjusted from 20 to 10% to explore the nitrogen removal performance of the reactor. The reactor was stopped for some time at the end of Phase II and subsequently restarted to observe the stability of PND process. In Phase III (days 75 ~ 142), the reactor was restarted and the number of days of operation was counted starting from 75th day. The operation conditions in Phase III were the same as those in Phase II.

### 2.3 Analytical methods and calculations

Sludge samples were regularly collected from the reactor and the concentrations of ammonia, nitrite and nitrate were determined according to standard methods (APHA, 2005). The dissolved NO/N_2_O in the reactor was detected online by Unisense NO/N_2_O-500 microelectrode (Supplementary Text S1). During the test, the microelectrode was directly placed into the biofilm reactor and dissolved NO was measured in-situ, with reading intervals of 10 s.

The total inorganic nitrogen (TIN) removal efficiency (TINRE) was calculated as shown below.


$$\text{TINRE}=\frac{{\mathrm{TIN}}_{\mathrm{Inf}}-{\mathrm{TIN}}_{\mathrm{Eff}}}{{\mathrm{TIN}}_{\mathrm{Inf}}}\times 100$$

Where, ${\mathrm{TIN}}_{\mathrm{Inf}}\;$ is the TIN of influent (mg-N/L), while ${\mathrm{TIN}}_{\mathrm{Eff}}\;$ is the TIN of effluent.

The simultaneous nitrification and denitrification (SND) efficiency (SNDE) was calculated (Third et al., 2003) as shown in the following equation:

SNDE = $(1-\frac{{{\mathrm{NO}}_{x}^{-}}_{\text{produced}}}{{{\mathrm{NH}}_{4}^{+}}_{\text{removal}}})\times 100.$

Where, ${{\mathrm{NO}}_{x}^{-}}_{\text{produced}}$ is the increase in $\;{\mathrm{NO}}_{x}^{-}\;$ ( ${\mathrm{NO}}_{2}^{-}$ + ${\mathrm{NO}}_{3}^{-}$ ) from the beginning to the end of aeration, mg-N/L; while ${{\mathrm{NH}}_{4}^{+}}_{\text{removal}}$ is the decrease in ammonia from the beginning to the end of aeration, mg-N/L.

### 2.4 Microbial community analysis

To analyze microbial diversity and community composition, sludge samples were collected from the inoculated sludge on day 0, as well as biofilm samples from SBBR during the stable operation period on day 135.

#### 2.4.1 DNA extraction

Genomic DNA was extracted using the OMEGA Soil DNA Kit (M5635-02, Omega Bio-Tek, Norcross, GA, United States) following the manufacturer’s protocol, then stored at −20 °C until analysis. DNA concentration and purity were assessed with a NanoDrop NC2000 spectrophotometer (Thermo Fisher Scientific, Waltham, MA, United States) and agarose gel electrophoresis.

#### 2.4.2 PCR amplification and sequencing

The V3-V4 region of the bacterial 16S rRNA gene was amplified using the primers 338F (5′-ACTCCTACGGGAGGCAGCA-3′) and 806R (5′-GGACTACHV GGGTWTCTAAT-3′), which included sample-specific 7-bp barcodes. PCR mixture consisted of 5 μL of 5 × buffer, 0.25 μL of Fast Pfu DNA Polymerase (5 U/μl), 2 μL of dNTPs (2.5 mM), 1 μL of each forward and reverse primer (10 μM), 1 μL of DNA template, and 14.75 μL of ddH_2_O. The amplicons were purified using Vazyme VAHTS™ DNA Clean Beads (Vazyme, Nanjing, China), quantified with the Quant-iT PicoGreen dsDNA Assay Kit (Invitrogen, Carlsbad, CA, United States), pooled in equal concentrations, and sequenced (2 × 250 bp paired-end) on an Illumina NovaSeq 6,000 platform (SP Reagent Kit, 500 cycles) by Shanghai Personal Biotechnology Co. Ltd. (Shanghai, China). All sequence raw data used in this study were deposited in the China National center for Bioinformation (CNCB) repository, accession number CRA033567.

#### 2.4.3 Sequence analysis

Raw reads were analyzed in QIIME2 (v. 2022.11) (Bolyen et al., 2018). Sequences were demultiplexed with the demux plugin, primers trimmed with cutadapt (Martin, 2011), then filtered, denoised, merged, and chimera-checked using DADA2 (Callahan et al., 2016). Non-singleton ASVs were aligned with MAFFT (Katoh et al., 2002) and used to build a tree via FastTree2 (Price et al., 2009). Alpha-diversity metrics (Chao1, Observed species, Shannon, Simpson, and Good’s coverage) were calculated using the diversity plugin. Taxonomic assignment was performed with the classify-sklearn naive Bayes classifier (Bokulich et al., 2018) against the SILVA 138.1 database (Koljalg et al., 2013).

#### 2.4.4 Bioinformatics and statistical analysis

Downstream analyses were completed in QIIME2 and R (version 3.2.0). ASV-level diversity indices and ranked abundance curves were used to assess richness and evenness across samples. Group differences in microbial composition were evaluated using PERMANOVA (McArdle and Anderson, 2001), ANOSIM (Clarke, 1993), and PERMDISP (Anderson et al., 2006). Taxonomic distributions were visualized using MEGAN (Huson et al., 2011) and GraPhlAn (Asnicar et al., 2015). Differentially abundant taxa were identified by LEfSe (Segata et al., 2011) and MetagenomeSeq (Zgadzaj et al., 2016), while supervised analyses including OPLS-DA (Mahadevan et al., 2008) and random forest (Breiman, 2011; Liaw and Wiener, 2002) were conducted to distinguish microbial variations among samples.

## 3 Results

### 3.1 Nitrogen removal performance of SBBR

**Figure 2:**
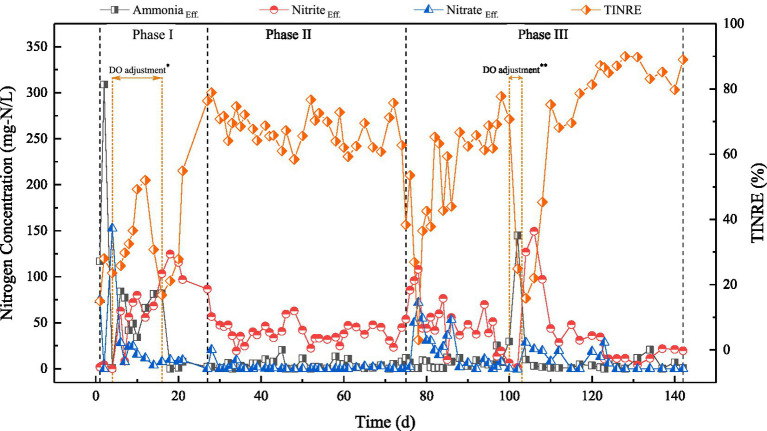
Profiles of ammonia, nitrite, nitrate concentrations and TINRE during the experimental periods with the different phases of SBBR indicated by lines. DO adjustment^*^: The aeration rate and mode were adjusted to ensure that the dissolved oxygen levels in the mixed liquor and at the biofilm surface remained as consistent as possible. DO adjustment^**^: The aeration duration was extended.

In this study, nitrogen removal performance of SBBR was investigated by observing the changes in the concentrations of ammonia, nitrite, nitrate, and TINRE during the treatment of ammonia-rich wastewater. The results have been shown in Figure 2.

The SBBR was operated for 142 days and the operation was divided into three phases. In Phase I (days 1 ~ 27), ammonia did not oxidize completely and the denitrification performance of reactor was poor. Considering these issues, the aeration rate and method were adjusted to ensure that DO levels in the mixed liquor and on the surface of the biofilm in the reactor were as consistent as possible, thereby resolving the issue of ammonia accumulation. This suggests that the appropriate DO concentration is the key to ensure the ammonia oxidation efficiency of high-strength ammonia wastewater. As shown in Figure 2, the products of nitrification process during aeration were nitrite and nitrate, which indicated the occurrence of partial nitrification in the initial phase of the SBBR. This may be due to the high concentration of ammonia in the influent, which led to the formation of FA-rich [up to 93.09 mg/L (Supplementary Figure S3)] environment in the reactor. This inhibited the activity of NOB, resulting in nitrite accumulation during the nitrification process.

Due to incomplete ammonia oxidation in Phase I, the drainage ratio was adjusted from 20 to 10% in Phase II (days 28 ~ 74) to reduce the ammonia-nitrogen load in the reactor. Due to nitritation, the main product in the effluent was nitrite, while nitrate was almost absent. In this phase, the average concentration of nitrite was 38.97 ± 10.61 mg-N/L, while the average TINRE was 67.88 ± 5.28%. This indicated nitrogen removal in the reactor through PND, with potential for long-term stable operation.

In Phase III (days 75 ~ 142), the reactor was stopped for a period and then restarted to investigate the stability of short-cut nitrogen removal from ammonia-rich wastewater. After restart of SBBR, TINRE was lower than that in Phase II. At the same time, nitrite and nitrate were present in the effluent, indicating the deterioration of partial nitrification process. As the operation proceeded, the nitrate concentration in effluent decreased and TINRE gradually increased, indicating the rapid continuation of PND for nitrogen removal. On day 100 of reactor operation, accumulation of ammonia occurred in the SBBR. To mitigate further accumulation, the aeration time was extended to facilitate the conversion of the accumulated ammonia into nitrite during day 100 to 103 of reactor operation. Afterward, the operating conditions of the SBBR remained consistent with the previous conditions. Despite experiencing a high ammonia load shock, SBBR maintained stable operation following a rapid recovery, with an average TINRE of 83.37% ± 6.93%, which was higher than that observed in phrase II.

In summary, the average TINRE of SBBR process was more than 80%, indicating the good nitrogen removal performance of PND at under C/N 5. During treatment of ammonia-rich wastewater, PND can occur quickly and spontaneously in SBBR. Considering the influence of substrate and DO concentration fluctuation, PND-SBBR showed good tolerance and can quickly restore the performance of system to ensure effective removal of pollutants. These results can serve as a reference for application of PND-SBBR process to treat ammonia-rich wastewater.

### 3.2 Changes in relevant indicators during the typical cycle of SBBR

#### 3.2.1 Changes in indicators at discharge ratio of 20%

**Figure 3:**
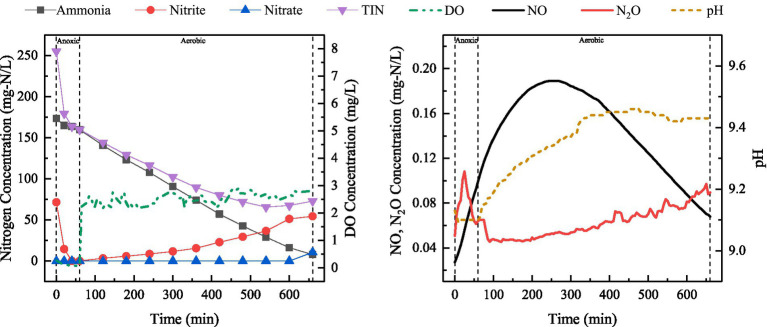
Variations in ammonia, nitrite, nitrate, TIN, DO, NO and N_2_O levels, as well as pH during a typical cycle (during stable operation in Phase I) at the discharge ratio of 20%.

The changes in the concentrations of ammonia, nitrite, nitrate, DO, TIN, dissolved NO and dissolved N_2_O were analyzed for the typical cycle of SBBR under discharge ratio of 20%. The results have been shown in Figure 3.

As shown in Figure 3, nitrite was used as the main electron acceptor for denitrification in the anoxic stage due to partial nitrification in the aerobic aeration stage of SBBR. At C/N 5, 71.58 mg-N/L of nitrite was reduced during anoxic stage, resulting in generation of NO and N_2_O as products. TIN concentration continued to decrease during aerobic stage. After 540 min, it basically remained stable, which may be related to the endogenous denitrification by microorganisms. At the end of a typical cycle, TIN in the effluent was 72.87 mg-N/L, while SNDE was 57.26%.

The NO concentration gradually increased after the beginning of cycle, reaching 0.19 mg-N/L at 265 min, and then began to decrease until the end of the cycle. The nitrogen conversion characteristics revealed that the pathways of NO production in anoxic and aerobic aeration stages were different. The NO concentration continued to increase at the beginning of aerobic stage, when only ammonia was present in the reactor. The NO production mainly started from the ammonia oxidation process. N_2_O level increased rapidly to 0.11 mg-N/L during anoxic stage and then declined, which corresponded to the changes in nitrite concentration. This may be due to the rapid reduction of remaining nitrite in the previous cycle through exogenous denitrification pathway. N_2_O concentration increased slowly during aerobic stage until the end of the cycle. Unlike conventional aerobic nitrification, which typically consumes alkalinity, this study observed an increase in pH during the aerobic phase (Figure 3). This phenomenon indicates that the oxygen gradient formed by the biofilm mediated the occurrence of SND. Under conditions of limited buffering capacity, the imbalance between alkalinity consumption and generation during SND resulted in a net increase in alkalinity (Supplementary Text S2). Furthermore, aeration during the aerobic phase facilitated the stripping of CO_2_ produced by denitrification, thereby shifting the carbonate system towards de-acidification and further enhancing alkalinity.

#### 3.2.2 Changes in indicators at drainage ratio of 10%

**Figure 4:**
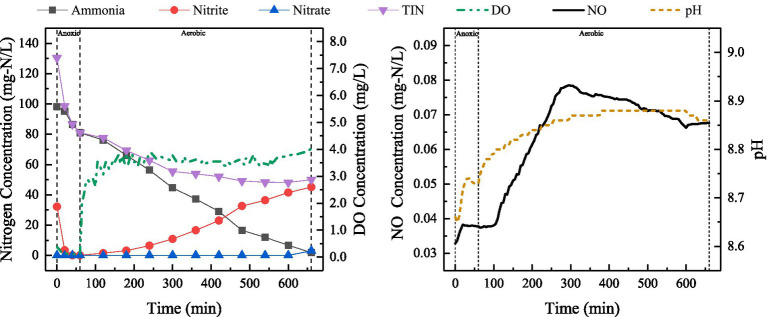
Variations in ammonia, nitrite, nitrate, TIN, DO and NO concentrations, as well as pH during a typical cycle (during stable operation in Phase II) at the discharge ratio of 10%.

After adjusting the reactor drainage ratio of 10%, a typical cycle was selected during stable operation and the changes in relevant indicators were examined. The results have been shown in Figure 4.

As shown in Figure 4, discharge ratio of SBBR was adjusted from 20 to 10%, i.e., initial ammonia concentration in the reactor was reduced. During anoxic stage, TIN removal mainly occurred through exogenous denitrification. During aerobic stage, ammonia oxidation products were dominated by nitrite. The profile in TIN at drainage ratio of 10% was similar to that at a drainage ratio of 20%, and the decrease in TIN lasted around 540 min. TIN concentration in the effluent (49.97 mg-N/L) was lower, indicating that the decrease in ammonia-nitrogen load not only led to a higher ammonia removal rate, but also to a better nitrogen removal performance. Meanwhile, the initial pH of the reactor was 8.7. During the anoxic phase, the pH first increased and then decreased. In the aerobic phase, the pH showed a slow upward trend, which was facilitated by oxygen transfer limitations within the biofilm structure that promoted SND.

A slight increase in NO was observed during anoxic stage, and this change basically corresponded to the change in nitrite concentration. During aerobic stage, NO initially increased and then decreased. The peak concentration of NO was 0.08 mg-N/L, which was significantly lower than that under 20% drainage ratio (0.19 mg-N/L). This indicated that NO production during aerobic stage was related to ammonia concentration, and it was mainly attributed to NH_2_OH oxidation at high ammonia-nitrogen load in SBBR. Previous studies have reported that NO production during biological nitrogen removal accounts for approximately 0.07–0.2% of the nitrogen load (Ahn et al., 2011; Kampschreur et al., 2008a). In activated sludge reactors employing PND for the treatment of ammonia-rich wastewater, NO concentrations ranged from 0.02 to 0.29 mg-N/L (Zhao J. et al., 2022), which is comparable to the levels of NO observed in this study.

#### 3.2.3 Changes in indicators in the typical cycle after SBBR restart

**Figure 5:**
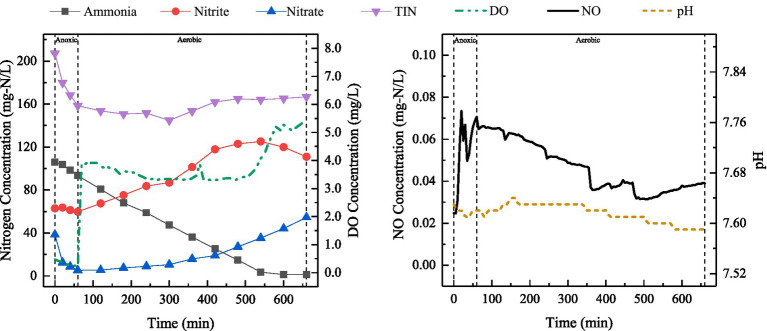
Variations in ammonia, nitrite, nitrate, TIN, DO and NO concentrations, as well as pH following the restart of the SBBR (initial stage of Phase III).

Figure 5 showed that the ammonia oxidation performance recovered well after SBBR restart and complete ammonia oxidization was achieved within 540 min. However, due to nitrate production during ammonia oxidation, the effluent contained both nitrite and nitrate at the end of the cycle. This led to denitrification during anoxic stage mainly using nitrate as an electron acceptor. In addition, the performance of SND was poor and did not recovered fully, resulting in higher concentration of TIN in the effluent. During aerobic stage, TIN concentration decreased slightly and remained almost stable after 300 min.

During anoxic stage of the typical cycle, NO concentration tended to rise, then fall and rise again (Figure 5). The initial rise in NO may be related to exogenous denitrification, with nitrate as the electron acceptor. With the decrease in nitrate concentration, NO concentration also decreased. The subsequent rise in NO concentration may be related to the presence of nitrite and insufficient electron donors. During the aerobic stage, NO concentration exhibited a gradual decline, which differed from the previously observed trend (Figure 4). This phenomenon may be attributed to the relatively high concentration of NO at the conclusion of the anoxic stage, coupled with a lower production rate of NO compared to its consumption rate during the aerobic stage. The pathways for NO consumption include reduction by denitrifying bacteria and aeration stripping. Compared with the previous typical operating cycle (Figure 4), nitrate production occurred during ammonia oxidation after the reactor was re-started, indicating the presence of NOB in the system. Competition for the substrate (nitrite) existed between nitrite oxidation and AOB denitrification process. Among them, NOB oxidizes nitrite to nitrate, whereas AOB denitrification reduces nitrite to NO. Therefore, the presence of NOB could be also one of the reasons for the relatively low NO production rate during the aerobic phase after re-startup of the reactor.

**Figure 6:**
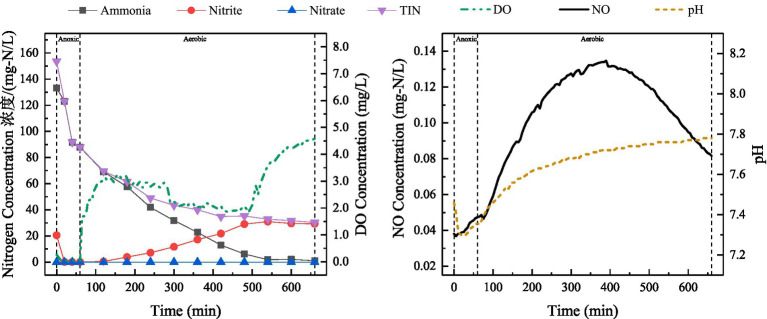
Variations in ammonia, nitrite, nitrate, TIN, DO and NO concentrations, as well as pH during the stable operation of the SBBR (during stable operation in Phase III).

Figure 6 demonstrated the variations in indicators in a typical cycle during stable operation of SBBR after restart. During the anoxic stage, complete nitrite removal was achieved through exogenous denitrification. During aerobic stage, complete ammonia oxidization was achieved at approximately 540 min with no nitrate production and PND process was resumed in the reactor. The decrease in TIN concentration also continued until 540 min, decreasing by 57.69 mg-N/L, indicating that the nitrite was mostly removed during aerobic stage. This finding reflected the good performance of simultaneous partial nitrification and denitrification (SPND). NO concentration first increased and then decreased, with a peak concentration of 0.13 mg-N/L during aerobic stage. The results showed that SNDE during nitrogen removal from high-strength ammonia wastewater was 66.42% (Figure 6), indicating the better SND effect of reactor. This suggested that nitrogen removal was carried out through SPND process with nitrite as an intermediate product. This helped in improving the efficiency of nitrogen removal and reducing energy consumption. These results indicate that SPND is a better biochemical treatment method for nitrogen removal from ammonia-rich wastewater.

### 3.3 Analysis of microbial diversity and community structure

To further understand the characteristics of microbial community in the SBBR during treatment of high-strength ammonia wastewater, Alpha diversity and relative abundance of microbial species in inoculated sludge and SBBR biofilm were determined.

#### 3.3.1 Microbial diversity analysis

Chao1, Shannon, Simpson and Coverage indices were used to characterize the diversity of microbes in the samples. Chao1 index characterizes the richness, while microbial diversity can be reflected by Shannon and Simpson indices. Higher Chao1 and Shannon indices represent the higher species diversity and richness, while higher Simpson index reflects lower richness and diversity of microbial species (Wang et al., 2021; Xu et al., 2018).

As shown in Table 1, both Chao1 and Shannon indices of microorganisms decreased in SBBR biofilm compared to the inoculating sludge, while the changes in Simpson index were not significant. This indicated the decrease in the abundance and diversity of microbial community during the stable operation of SBBR, which may be related to the reduced complexity of reactor operation. The inoculating sludge was procured from a nitrogen and phosphorus removal reactor. However, the present experiment mainly focused on nitrogen removal, which resulted in a more concentrated distribution of microorganisms. In addition, Coverage index values in both the inoculating sludge and SBBR biofilm were greater than 0.99, indicating that the Coverage index met the requirements and well reflected the actual microbial community in the samples.

**Table 1:** Alpha diversity of microbial community.

Sample types	Chao1	Shannon	Simpson	Coverage
Inoculated sludge	2263.36	8.25	0.98	0.99
SBBR biofilm	568.99	5.50	0.94	0.99

As shown in Supplementary Figure S7, OTU rank range of inoculating sludge on the horizontal axis was much larger than that of the SBBR biofilm, suggesting the decrease in the species richness and homogeneity in SBBR. The very steep curve of biofilm samples during the stable operation of SBBR suggests that the number of dominant strains in the SBBR was much larger, with a lower diversity and a more concentrated distribution of microbial species.

#### 3.3.2 Microbial community structure

**Figure 7:**
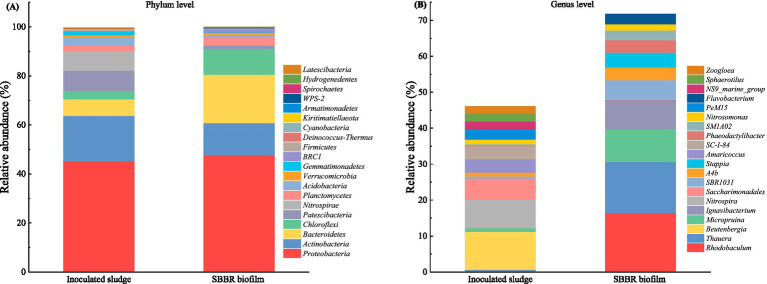
Relative abundance of microorganisms at the phylum level **(A)** and genus level **(B)** in the inoculated sludge and SBBR biofilm.

As shown in Figure 7A, the dominant bacterial phyla in the inoculating sludge were mainly *Proteobacteria* (45.09%), *Actinobacteria* (18.66%), *Patescibacteria* (8.24%), *Nitrospirae* (7.91%), and *Bacteroidetes* (6.78%), *Chloroflexi* (3.42%), *Acidobacteria* (3.1%), *Planctomycetes* (2.38%) and others. In a previous study conducted on microbial communities during wastewater treatment, *Proteobacteria*, *Bacteroidetes* and *Actinobacteria* were observed as the dominant bacterial phyla with higher abundance in activated sludge systems, whereas *Chloroflexi*, *Acidobacteria* and *Planctomycetes* had lower abundance (Wang et al., 2021), which is consistent with the findings of this study.

The main microbial phyla in SBBR biofilm were similar to the inoculating sludge (Figure 7A). Abundances of *Proteobacteria* (47.6%), *Bacteroidetes* (19.71%), Actinobacteria (13.23%), *Chloroflexi* (10.59%), and *Planctomycetes* (3.53%) in biofilm were higher compared to that in the inoculating sludge. *Proteobacteria* had the highest relative abundance and was dominant in the microbial community of SBBR biofilm. Bacteria of *Proteobacteria* phylum have been reported to play an important role in nitrogen removal under microaerobic conditions during SND-SBBR process (Tong et al., 2019).

As shown in Figure 7B, the main bacterial genera in inoculating sludge were *Nitrospira*, *Nitrosomonas*, and *NS9_marine_group*, which are known for their involvement in nitrification and denitrification. Among them, *Nitrospira* and *Nitrosomonas* are NOB and AOB, which are commonly found in wastewater treatment plants. These genera have also been observed as functional genera in nitrification reactors in previous studies (Zhao et al., 2022a). *NS9_marine_group* has also been reported to have denitrification function (Liu et al., 2022). Thus, functional microorganisms with nitrogen metabolism capacity were already present in the inoculating activated sludge.

The main microbial genera in SBBR biofilm included *Rhodobaculum* (16.51%), *Thauera* (14.11%), *Ignavibacterium* (8.07%), *Stappia* (4.09%), *Phaeodactylibacter* (3.46%), *Flavobacterium* (2.74%) and *Nitrosomonas* (1.71%), among others (Figure 7B). *Nitrospira*, *Thauera*, *Nitrosospira*, *Ignavibacterium*, and *Nitrosomonas* have also been observed as main genera in activated sludge system in a previous study (Wang et al., 2021), which is consistent with the findings of this study. In summary, during the stable operation of the SBBR, *Thauera*, *Stappia*, and *Nitrosomonas* were markedly enriched, indicating that these genera may play essential roles in PND process. However, this study involved only a single sampling event during the stable operational period, which represents a limitation as it could not capture the dynamic changes in the microbial community. Future research should include multi-time-point sampling and functional gene analyses to further elucidate the roles and mechanisms of the associated microorganisms.

## 4 Discussion

### 4.1 Nitrogen removal performance of SBBR for treatment of high-strength ammonia wastewater

SBBR-based PND could better cope with substrate fluctuations, changes in DO concentration, and high ammonia load, mainly due to the strong adaptability of biofilm to the changes in wastewater quality and quantity, its ability to withstand shock loads (Wang et al., 2000) and the microbial community structures. The changes in TIN concentration shown in Figures 3, 4, 6 indicated that most of the nitrite removal in SBBR occurred during aeration stage. The nitrogen removal during aerobic stage was accomplished through SPND, with SNDE reaching 66.42% (Figure 6).

On the one hand, functional microbial groups played a crucial role in the PND process. Although aerobic denitrifying bacteria such as *Pseudomonas* and *Thiosphaera pantotropha* were not detected in an SBBR (Robertson and Kuenen, 1984; Wang et al., 2021), dominant phyla *Proteobacteria* and *Bacteroidetes*, both key participants in SND, were present. *Proteobacteria*, which made up the highest relative abundance in the SBBR of this study, include a wide range of metabolic types, and both nitrifying and denitrifying bacteria belong to this phylum (Jiang et al., 2019; Racz et al., 2010; Tong et al., 2019; Xiao et al., 2015). Bacteroidetes are crucial for achieving SND and it has been observed as a dominant bacteria of phylum level in nitrification reactors (Ramirez-Vargas et al., 2015), with important roles in organic matter degradation (Qiu, 2020). At the genus level, although *Thauera* was not a dominant genus in inoculating sludge, its high relative abundance in SBBR biofilm indicated the strong denitrification activity of SBBR (Qiu, 2020; Scholten et al., 1999). This is attributed to the anoxic phase of the SBBR and the anoxic environment within the biofilm, both of which promote the enrichment of T*hauera*. *Thauera* demonstrates a high denitrification potential in reactors that operate under alternating anoxic and oxic conditions. In addition, *Stappia* was also identified as one of the dominant genera during denitrification (He et al., 2021). When aerobic stage is required to undertake part of the nitrogen removal or when pronounced low DO levels exist in the system, *Stappia*, known for its aerobic denitrification capabilities, occupies a favorable ecological niche and assumes a prominent role. The enrichment of these functional microbial groups helps explain the high SNDE (66.42%) observed in this study.

On the other hand, in the treatment of nitrogen-containing wastewater using biofilm technology, the mass transfer and diffusion limitations imposed by biofilm thickness result in a gradient change in DO concentration from the biofilm surface to its interior (Zhang et al., 2016). The aerobic layer on the biofilm surface supports the activity of aerobic microorganisms, such as AOB, while the inner layer of the biofilm provides microaerobic or anoxic environments for denitrifying microorganisms such as *Thauera* and *Stappia*. In addition, the denitrification during the aerobic stage may utilize intracellularly stored organic carbon or storage polymers (e.g., polyhydroxyalkanoates), enabling the system to maintain the SND efficiency even under conditions where external organic carbon supply is insufficient.

In summary, PND not only shortens the pathway of biological nitrogen removal but also maximizes the utilization of the in-situ carbon sources in wastewater under carbon-limited conditions, thereby alleviating the common issue of insufficient carbon sources in wastewater treatment. The revealed synergistic relationship between microbial community composition and biofilm mass-transfer characteristics offers both theoretical and practical insights for optimizing the performance of biofilm-based processes in treating ammonia-rich wastewater.

### 4.2 Mechanism of partial nitrification occurrence during treatment of high-strength ammonia wastewater

Partial nitrification could be achieved *in situ* in ammonia-rich environments without requiring any external inhibitor or changes in operating conditions. Figure 6 showed that SBBR lost its ability to oxidize nitrite to nitrate during stable operation. In the nitrification process, AOB such as *Nitrosomonas* are primarily responsible for converting ammonia to nitrite, while NOB such as *Nitrospira* convert nitrite to nitrate. However, in this study, *Nitrospira* was absent from the top 20 genera in the microbial community of SBBR biofilm samples, whereas in the inoculated sludge its relative abundance reached 7.91%, making it one of the dominant genera, which indicates that *Nitrospira* was strongly inhibited or even completely eliminated during SBBR operation. This may be due to the high concentrations of ammonia and nitrite accumulation, which leads to the synergistic inhibition of NOB, such as *Nitrospira*, growth by FA and FNA (Duan et al., 2019; Sui et al., 2016). Consequently, SBBR spontaneously created conditions for PND without implementation of other regulatory strategies.

The NO also strongly inhibits the NOB. In a previous study, NO reversibly inhibited *Nitrobacter*, which had a low affinity for nitrite (high Ks), and strongly inhibited *Nitrospira*, which had a high affinity for nitrite (low Ks) (Courtens et al., 2015). In the activated sludge reactor, NO inhibits the activity of NOB by inducing toxic effects after contact with NOB, resulting in rapid occurrence of partial nitrification (Zhao et al., 2022a). In this study, NO production was observed throughout the typical cycle. NOB were synergistically inhibited by FA (up to 93.09 mg/L) and NO (up to 0.19 mg-N/L) in the initial phase (Phase I, days 1–27) of SBBR operation, which accelerated the occurrence of partial nitrification. As nitrite accumulated, the synergistic effects of FNA (0.012 mg/L) and NO led to stable operation of PND. Especially, when DO concentration was 2 ~ 3 mg/L and ammonia feeding was lower, partial nitrification also maintained long-term stable operation. Therefore, focusing on NO production mechanism that inhibits microorganisms is very important to reveal the reasons for occurrence of partial nitrification.

### 4.3 NO production mechanisms

Production and accumulation of NO were observed during anoxic stage, which was different from conventional denitrification. NO accumulation generally does not occur due to high NO reduction rate (higher than nitrite reduction rate) (Zhou et al., 2011). According to the study by (Wang et al., 2019a), the accumulation of NO during denitrification is contingent upon the rate of nitrite reduction. Specifically, NO accumulation occurs when the rate of NO reduction is lower than that of nitrite reduction. In this study, decrease in nitrite concentration corresponded to the increase in NO concentration at the beginning of anoxic stage (Figure 4), which may be attributed to rapid reduction of nitrite under carbon source limitation. Electron competition leads to higher nitrite reduction rate than NO reduction rate, resulting in an imbalance between NO production and consumption. In addition, NO can cause changes in genetic material and adversely affect the microorganisms (Schulthess et al., 1995; Wang et al., 2016). NO accumulation during denitrification is also associated with its own toxic effects. For instance, NO inhibits *Nor* (Wang et al., 2023), which prevents further NO reduction.

**Figure 8:**
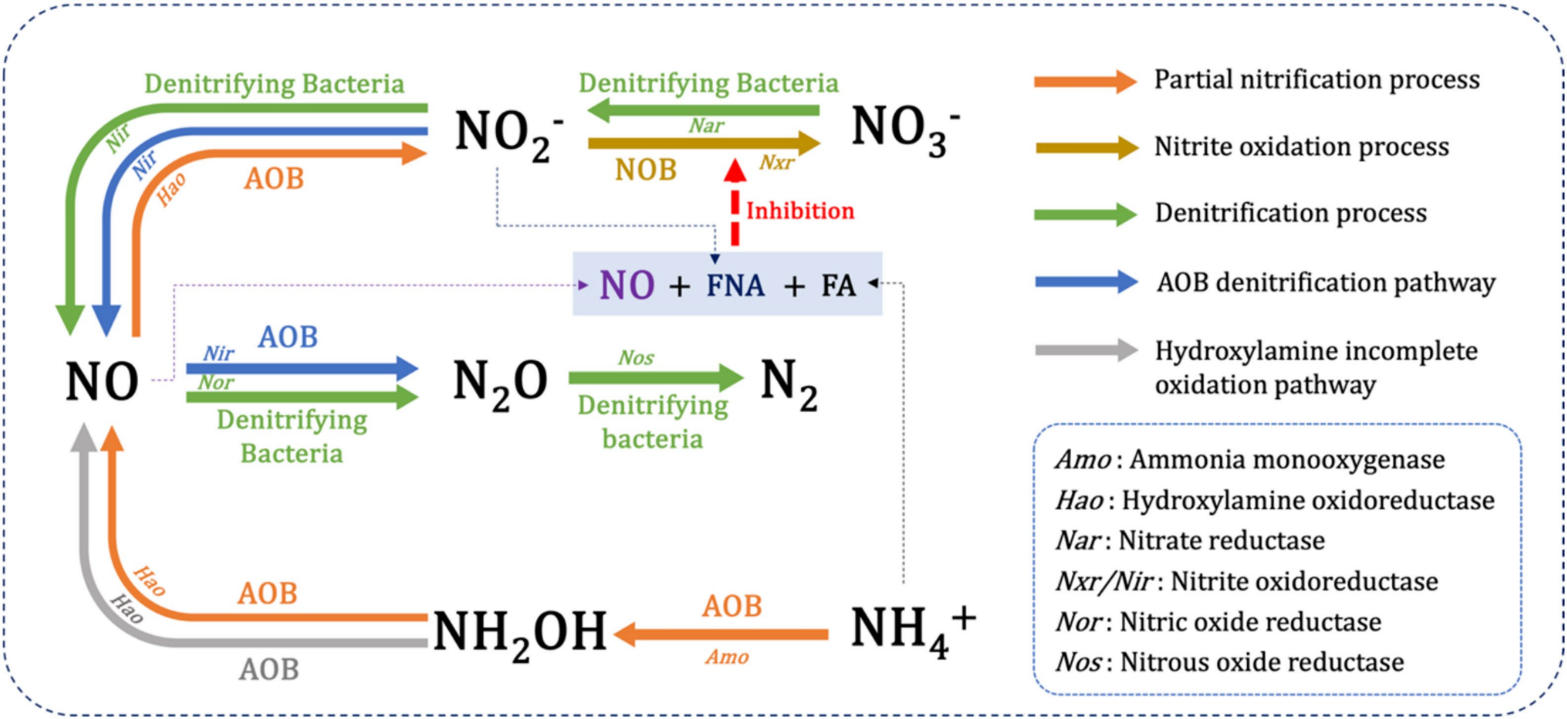
NO production mechanism during short-cut nitrogen removal from high-strength ammonia wastewater in a sequencing batch biofilm reactor.

The NO accumulation also occurred during aerobic aeration in SBBR. The maximum NO concentration in the typical cycle was 0.19 mg-N/L, and its source was incomplete oxidation of NH_2_OH due to high ammonia-nitrogen load and AOB denitrification (Figure 8). Firstly, a transient accumulation of NH_2_OH was noticed during ammonia oxidation, with concentrations ranging from 0.003 to 4.3 mg-N/L (Soler-Jofra et al., 2021). NO is usually considered a by-product of the incomplete oxidation of NH_2_OH (Ni et al., 2014; Zhu et al., 2013). NO has been reported as a specific intermediate during the oxidation of NH_2_OH to nitrite, catalyzed by hydroxylamine oxidoreductase (HAO) (Caranto and Lancaster, 2017). Therefore, incomplete oxidation of NH_2_OH during treatment of ammonia-rich wastewater is one of the sources of NO, with N_2_O being the product of NO reduction (Stein, 2011). Secondly, accumulated nitrite partially replaces O_2_ as an electron acceptor. AOB reduces nitrite sequentially to NO and N_2_O via copper-containing nitrite reductase (*NirK*) and heme NO reductase (*Nor*) (Law et al., 2013; Zhu et al., 2013), thereby generating N_2_O as the final product (Ni et al., 2013). Nitrite accumulation increases the contribution of AOB denitrification pathway to NO and N_2_O, which may be attributed to higher expression of *NirK* gene at high concentrations of nitrite (Beaumont et al., 2004). However, overexpression of *Nir* genes may also lead to lower expression of genes encoding NO reduction-related enzymes, which further contributes to NO accumulation (Kampschreur et al., 2008b; Kester and Wietse, 1997).

The NO is produced during ammonia oxidation usually due to synergistic contributions of multiple pathways of NH_2_OH oxidation and AOB denitrification. The mechanisms regulating the pathways of NO and N_2_O production are associated with the changes in the availability of electron donors and acceptors, which cause an imbalance between electron production and consumption. In the reactor, O_2_ acts as an electron acceptor and competes with nitrite, resulting in a slower rate of AOB denitrification, which further promotes the oxidation of NH_2_OH. With the depletion of electron donors, concentration of electron acceptors decreases. This leads to lack of electrons to activate AOB denitrification pathway, which increases NO production through NH_2_OH oxidation pathway (Anderson et al., 1993; Peng et al., 2015; Perez-Garcia et al., 2014).

In this study, N_2_O concentrations were measured only for a set of typical cycle (Figure 3). At C/N 5, the maximum level of N_2_O reached 0.11 mg-N/L during anoxic stage in the SBBR, which might be related to the inhibition of *Nos* by high concentrations of nitrite and NO, as well as the electron competition. Only limited N_2_O accumulation was observed in the aerobic stage of SBBR. Studies have shown that high concentrations of nitrite and low levels of DO (<1 mg/L) are the primary drivers of N₂O production via AOB denitrification during aerobic nitrification (Chen et al., 2020). In this study, DO concentration during aerobic stage was consistently maintained above 2 mg/L. Under these high-DO conditions, AOB denitrification pathway was significantly suppressed, even in the presence of certain levels of nitrite. This observation aligns with the findings of Massara et al. (2018), who reported that when DO levels exceeded 1.8 mg/L, the contribution of AOB denitrification to N₂O became negligible. Furthermore, to minimize N₂O generation in wastewater treatment, it is essential to enhance aerobic denitrification while selectively suppressing AOB denitrification (Hao et al., 2023). The occurrence of SPND during aerobic aeration in the SBBR was likely a key factor contributing to the relatively low N₂O concentrations observed in this study. pH also reasonably contributes to the reduction in N_2_O production during biological nitrogen removal process. In the SND process, a lower pH is more conducive to N_2_O production. On the contrary, higher pH leads to lower N_2_O emission. Studies have demonstrated that maintaining the pH within the range of 6.8–8.0 results in negligible N₂O generation in the system (Thörn and Sörensson, 1996). Zhu et al. (2010) investigated the influence of pH on N₂O emissions during the nitrification process in a sequencing batch reactor system. Their results indicated that N₂O emissions gradually decreased as pH increased from 6 to 8, reaching a minimum at pH = 8. In summary, during treatment of ammonia-rich wastewater by PND process, attention should be paid to the generation of by-products, such as NO and N_2_O to understand the production and emission of NO and N_2_O during biological nitrogen removal, as well as to develop strategies for resolving the environmental problems caused by the by-products.

## 5 Conclusion

This study investigated the nitrogen removal performance and the role of NO in the short-cut nitrogen removal process for treating high-strength ammonia wastewater. Under a C/N ratio of 5, SBBR achieved an average TINRE of 83.37 ± 6.93%, while exhibiting stable SPND performance with an efficiency of 66.42%. During reactor operation, NO production was predominantly observed in the aerobic phase, where the maximum NO concentration reached 0.19 mg-N/L. NO primarily originated from the incomplete oxidation of NH_2_OH due to high ammonia loading and AOB denitrification. Meanwhile, synergistic inhibition of NOB by NO, FA and FNA contributed to rapid spontaneous occurrence of PND, which resulted in stable operation of SBBR for a long time. In addition, the distribution of microbial communities was more concentrated in SBBR biofilm, as compared to inoculating sludge. *Thauera, Stappia* and *Nitrosomonas* played a vital role in the process of PND. In conclusion, this study provides valuable insights into the role of NO and its generation mechanism in the treatment of high-strength ammonia wastewater, which is essential for improving wastewater treatment technologies.

## Data Availability

The data presented in the study are deposited in the China National center for Bioinformation (CNCB) repository, accession number CRA033567: https://ngdc.cncb.ac.cn/gsa/browse/CRA033567.
