# Supplementary material for: Short-cut nitrogen removal from high-strength ammonia wastewater in a sequencing batch biofilm reactor: roles of NO and its production mechanism
Source: Front Microbiol. 2025 Dec 4;16:1653308. doi: 10.3389/fmicb.2025.1653308 (PMC12712650; doi:10.3389/fmicb.2025.1653308)
Supplement: Supplementary file 1 [file Supplementary_file_1.docx]

**Supplementary Material**

Short-cut nitrogen removal from high-strength ammonia wastewater in a sequencing batch biofilm reactor: roles of NO and its production

Junkai Zhao ^a, b^, Ju Zhang ^c,^ ^d^, Heng Yu ^a, b^, Wenjuan Yang ^c,^ ^d^, Jianqiang Zhao ^c,^ ^d,^ ^*^, Shuhan Lei ^c^, Jie Yang ^e^

*^a^ College of Chemistry and Chemical Engineering, Xi’an Shiyou University, Xi’an 710065, Shaanxi, China.*

*^b^ Shaanxi Province Key Laboratory of Environmental Pollution Control and Reservoir Protection Technology of Oilfields, Xi’an 710065, Shaanxi, China.*

*^c^ School of Water and Environment, Chang’an University, Xi’an 710064, Shaanxi, China.*

*^d^ Key Laboratory of Subsurface Hydrology and Ecological Effect in Arid Region (Chang’an University), Ministry of Education, Xi’an 710064, Shaanxi, China.*

*^e^ Shaanxi Anliansheng Environmental Protection Technology Co., Ltd., Xi’an 710064, Shaanxi, China.*

*^*^Corresponding author, E-mail address:* [*626710287@qq.com*](mailto:626710287@qq.com) *(J. Zhao).*

**List of Text, Figure and Table Captions**

**Text S1.** Determination of NO and N_2_O concentrations.

**Text S2.** Explanations for pH variations during aerobic stage of SBBR.

**Fig. S1** Standard Curves and Performance of Linear Regression of NO.

**Fig. S2** Standard Curves and Performance of Linear Regression of N_2_O.

**Fig. S3~S6** Variations in FA and FNA concentrations during typical cycles at different operational phrase of reactor.

**Fig. S7.** Rank abundance curves of microorganisms in the SBBR biofilm and inoculated sludge.

**Fig. S8** Oxygen mass transfer and diffusion characteristics in the biofilm.

**Table S1.** The composition of trace elements in synthetic wastewater.

**Text S1.** Determination of NO and N_2_O concentrations

**Detection Limit and Accuracy Range**

The limits of detection for the Unisense NO-500 and Unisense N₂O-500 microelectrodes were 1 nM and 0.1 µM, respectively, with an accuracy of ±1–3 % under standard conditions.

**Preparation of Standard curves of NO and N_2_O**

(1) Blow nitrogen at a rate of 5 L/min for more than 5 min to drive out 99.99% of NO/N_2_O in the water and make the solution of zero calibration for use.

(2) Aerate NO/N_2_O into pure water, and inflate continuously at a rate of 5 L/min for 15-20 min until it reaches saturation (the temperature must be kept constant 30°C during the inflation process, otherwise the saturation solubility will change.), spare. Saturated solubility can be obtained by looking up the table.

(3) Place NO/N_2_O microelectrode in the solution of zero calibration and read after the signal is stable.

(4) Take a certain volume of NO/N_2_O saturated solution and put it into a certain volume of nitrogen-filled solution, shake it gently to mix it, then place the microelectrode in the diluent until the signal stabilizes, read the value, and so on.

(5) Plot or use a linear regression to obtain a standard curve.

**The calibration Procedure for the N₂O Microelectrode is as follows:**

1. Polarization (Ensuring Baseline Stability)

(1) Insert the N₂O microelectrode into the PA channel socket and set the polarization voltage to –1300 mV for 10 minutes. Subsequently, adjust the voltage to –800 mV and immerse the electrode in deoxygenated water for polarization.

(2) The polarization duration should exceed 12 hours.

(3) Ensure that the baseline signal of the N₂O electrode remains below 20 mV prior to calibration.

2. Preparation of Calibration Solutions

(1) Zero Calibration Solution: Purge ultrapure water with high-purity nitrogen (or argon) at a flow rate of 5 L/min for over 5 minutes to remove dissolved N₂O. The treated solution will serve as the zero-point calibration solution.

(2) N₂O-Saturated Water: Bubble N₂O gas from a gas cylinder into ultrapure water at a flow rate of 5 L/min for 15–20 minutes, or until saturation is achieved. Maintain a constant temperature during bubbling to prevent variations in saturation solubility. The saturated concentration of N₂O can be determined using published solubility tables.

3. Calibration Steps

(1) Zero-Point Reading: Place the N₂O microelectrode in the zero-calibration solution; once the signal stabilizes, record the baseline voltage.

(2) Preparation and Measurement of Diluted Standards: Add appropriate volumes of N₂O-saturated water to known volumes of nitrogen-purged water, mix gently, and measure the electrode response after stabilization of the signal. For instance, at 20 °C, the saturated N₂O concentration in water is approximately 27 mM (27,000 µM). Mix 0.25, 0.50, 0.75, 1.00, and 1.25 mL of saturated N₂O solution with 250 mL of ultrapure water to obtain diluted standards of approximately 27, 54, 81, 108, and 135 µM, respectively.

**Text S2. Explanations for pH variations during aerobic stage of SBBR**

Theoretically, partial nitrification (NH_4_^+^ + 1.5 O_2_ → NO_2_^−^ + 2 H^+^ + H_2_O) produces 2 moles of H^+^, whereas denitrification using nitrite as the electron acceptor (8 NO_2_^−^ + C_6_H_12_O_6_ + 8 H^+^ → 4 N_2_ + 6 CO_2_ + 10 H_2_O) results in a net consumption of H^+^ by a factor of four. Consequently, under limited buffering capacity, the system pH tends to increase overall. When partial nitrification and denitrification proceed simultaneously, the acid–base equivalents produced and consumed do not offset each other, leading to a net increase in alkalinity.

**Fig. S1** Standard Curves and Performance of Linear Regression of NO


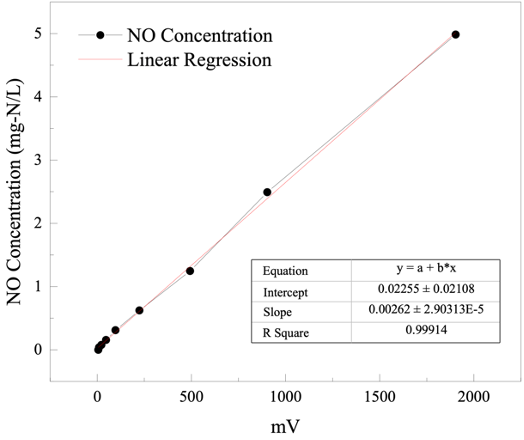


**Fig. S1 Standard Curves and Performance of Linear Regression of NO**

**Fig. S2** Standard Curves and Performance of Linear Regression of N_2_O


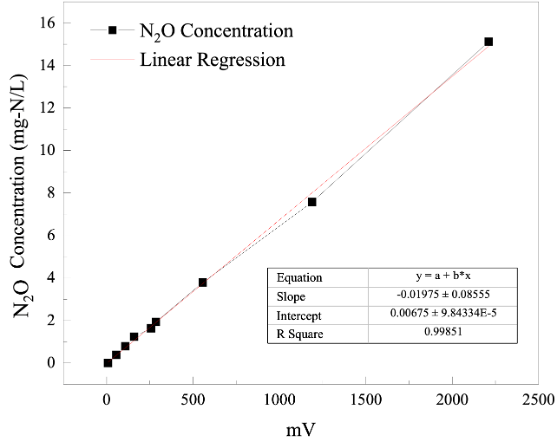


**Fig. S2 Standard Curves and Performance of Linear Regression of N_2_O**

**Fig. S3~S6** Variations in FA and FNA concentrations during typical cycles at different operational phrase of reactor.

To further clarify the influence of FA and FNA concentration dynamics on the establishment and stable operation of partial nitrification and denitrification (PND), FA and FNA concentrations were calculated for the typical cycles shown in Fig. 3, 4, 5, and 6. The calculation results are provided in Figures S3~S6.

Calculation formula for FA and FNA mass concentrations:

$\text{FA}\text{ }\text{=}\text{ }\frac{\text{17}}{\text{14}}\text{ }\text{×}\text{ }\frac{\text{C}_{\text{NH}_{\text{4}}^{\text{+}}\text{-}\text{N}}\text{ }\text{×}\text{ }\text{10}^{\text{pH}}}{\text{10}^{\text{pH}}\text{ }\text{+}\text{ }\text{e}^{\frac{\text{6344}}{\text{273+T}}}}$ （1）

Where FA represents the free ammonia concentration, mg/L；$\text{C}_{\text{NH}_{\text{4}}^{\text{+}}}$is the ammonia concentration, mg-N/L；T is the temperature, °C；and pH is the measured pH value.

$\text{FNA}\text{ }\text{=}\text{ }\frac{\text{46}}{\text{14}}\text{ }\text{×}\text{ }\frac{\text{C}_{\text{NO}_{\text{2}}^{\text{-}}\text{-N}}}{\text{10}^{\text{pH}}\text{ }\text{×}\text{ }\text{e}^{\frac{\text{-2300}}{\text{273+T}}}}$ （2）

Where FNA represents the free nitrous acid concentration, mg/L；$\text{C}_{\text{NO}_{\text{2}}^{\text{-}}}$is the nitrite concentration, mg-N/L；T is the temperature, °C；and pH is the measured pH value.

**Fig. S3 Variations in FA and FNA during a typical cycle (During stable operation in Phase I) at the discharge ratio of 20%.**

**Fig. S4 Variations in FA and FNA during a typical cycle (During stable operation in Phase II) at the discharge ratio of 10%.**

**Fig. S5 Variations in FA and FNA following the restart of the SBBR (initial stage of Phase III).**

**Fig. S6 Variations in FA and FNA during the stable operation of the SBBR (During stable operation in Phase III).**

**Fig. S7. Rank abundance curves of microorganisms in the SBBR biofilm and inoculated sludge.**


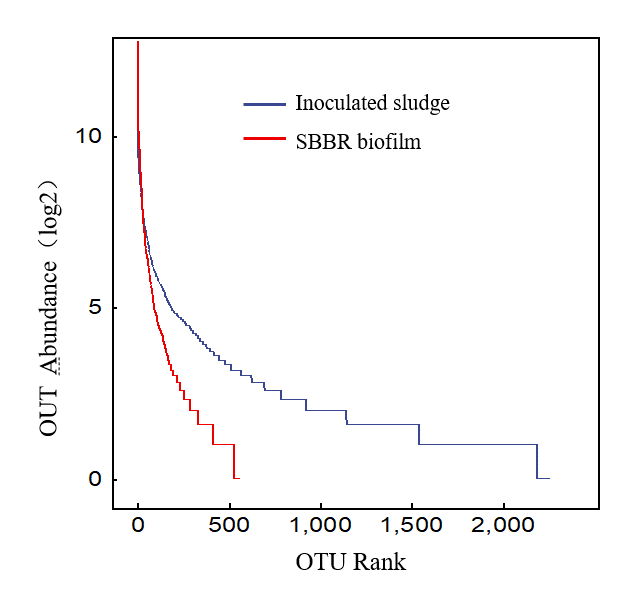


**Fig. S7. Rank abundance curves of microorganisms in the SBBR biofilm and inoculated sludge.**

**Fig. S8** Oxygen mass transfer and diffusion characteristics in the biofilm

**
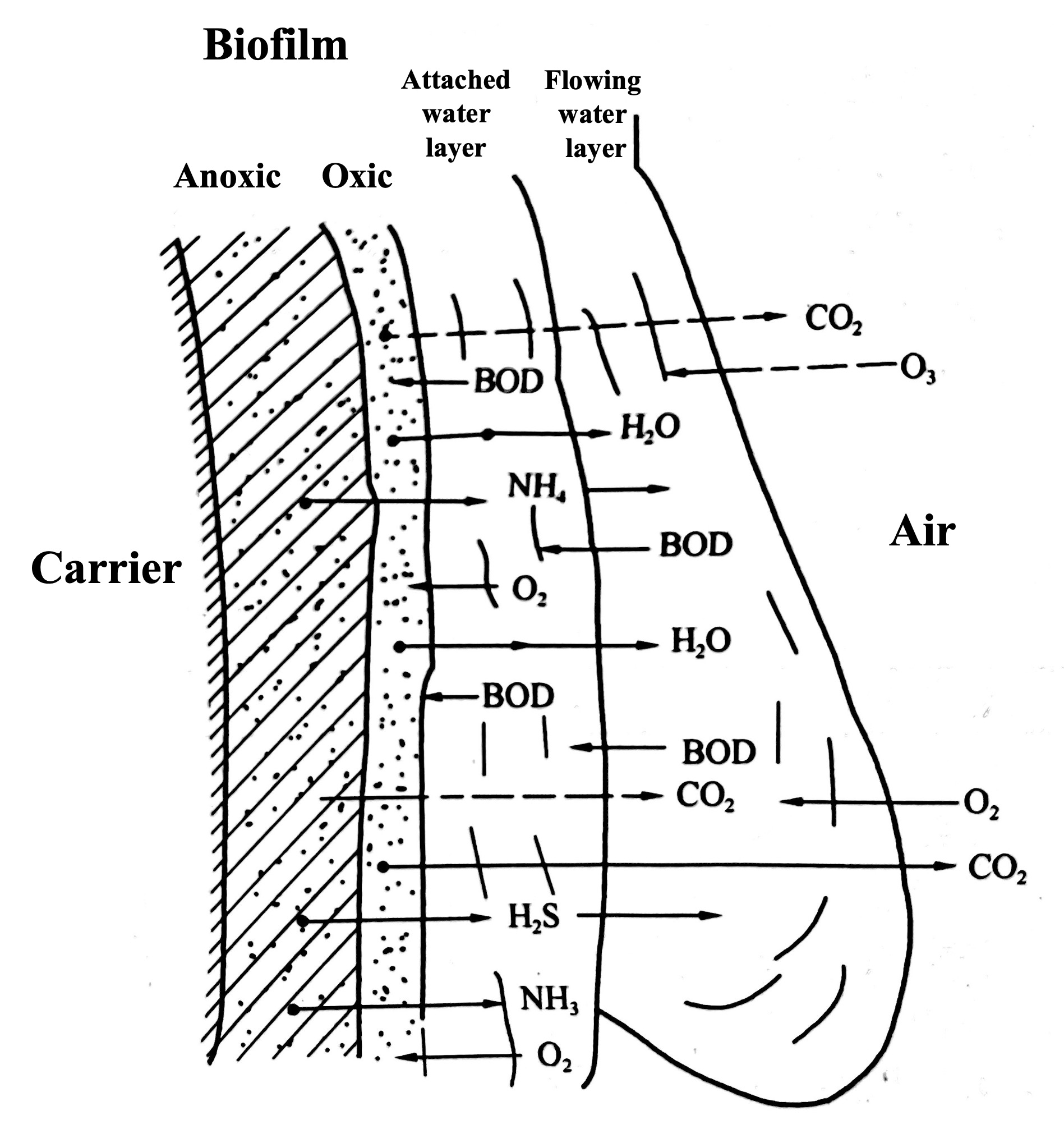
**

**Fig. S7 Oxygen mass transfer and diffusion characteristics during the treatment of high‑strength ammonia wastewater in a sequencing batch biofilm reactor**

**Table S1. The composition of trace elements in synthetic wastewater**

| Trace element compositions | Concentration |
| --- | --- |
| H_3_BO_3_ | 50 mg/L |
| CuCl_2_ | 30 mg/L |
| ZnCl_2_ | 50 mg/L |
| (NH_4_)_6_Mo_7_O_2_·4H_2_O | 50 mg/L |
| MgSO_4_·7H_2_O | 500 mg/L |
| CoCl_2_·6H_2_O | 50 mg/L |
| AlCl_3_ | 50 mg/L |
| NiCl_2_ | 50 mg/L |
| Concentrated hydrochloric acid | 1 ml/L |
